# A split intein T7 RNA polymerase for transcriptional AND-logic

**DOI:** 10.1093/nar/gku884

**Published:** 2014-09-27

**Authors:** Yolanda Schaerli, Magüi Gili, Mark Isalan

**Affiliations:** 1 EMBL/CRG Systems Biology Research Unit, Centre for Genomic Regulation (CRG), Dr. Aiguader 88, 08003 Barcelona, Spain; 2 Universitat Pompeu Fabra (UPF), Barcelona, Spain; 3 Department of Life Sciences, Imperial College London, London SW7 2AZ, UK

## Abstract

Synthetic biology has developed numerous parts for building synthetic gene circuits. However, few parts have been described for prokaryotes to integrate two signals at a promoter in an AND fashion, i.e. the promoter is only activated in the presence of both signals. Here we present a new part for this function: a split intein T7 RNA polymerase. We divide T7 RNA polymerase into two expression domains and fuse each to a split intein. Only when both domains are expressed does the split intein mediate protein trans-splicing, yielding a full-length T7 RNA polymerase that can transcribe genes via a T7 promoter. We demonstrate an AND gate with the new part: the signal-to-background ratio is very high, resulting in an almost digital signal. This has utility for more complex circuits and so we construct a band-pass filter in *Escherichia coli*. The split intein approach should be widely applicable for engineering artificial gene circuit parts.

## INTRODUCTION

As our ability to build more complex synthetic circuits improves, we also require new parts to build them (1,2). For example, rather few parts have been described that can be employed to build a transcriptional AND gate in *Escherichia coli*(3-8). The challenge here is to find or engineer a promoter that is only activated in the presence of two transcription factors—a setup hardly found in natural prokaryotic gene regulation. A possible way around this problem is to use a promoter that responds to a single activator, but where the activator consists of two essential parts whose expression can be controlled individually. For example, Moon *et al.* employed transcription factors whose activity is dependent on a chaperone (6). Alternatively, Shis and Bennett recently reported the use of split T7 RNA polymerase (T7 RNAP) (8). They took advantage of the observation that T7 RNAP can be divided between amino acids 179 and 180 and remains functional despite being formed from two subunits. The resulting non-covalently assembled polymerase does however have reduced activity and decreased processivity, relative to the native form (9).

Using split inteins is a way of covalently joining two parts of a protein (10). Fusing split inteins to two different protein domains leads to an auto-catalytic excision of the inteins, thereby joining the two protein domains (the exteins) into a full-length protein, via a peptide bond. This technology has been applied, for example, to protein tagging, to protein purification (11) and to transcription factors in mammalian cells in order to build logic gates (12,13). Here we generate a split intein T7 RNAP that self splices to form a native T7 RNAP. We show its functionality and utility in a transcriptional AND gate as well as in a more complex circuit that functions as a band-pass filter in *E. coli*.

## MATERIALS AND METHODS

### Network scaffold

**Figure 1.**
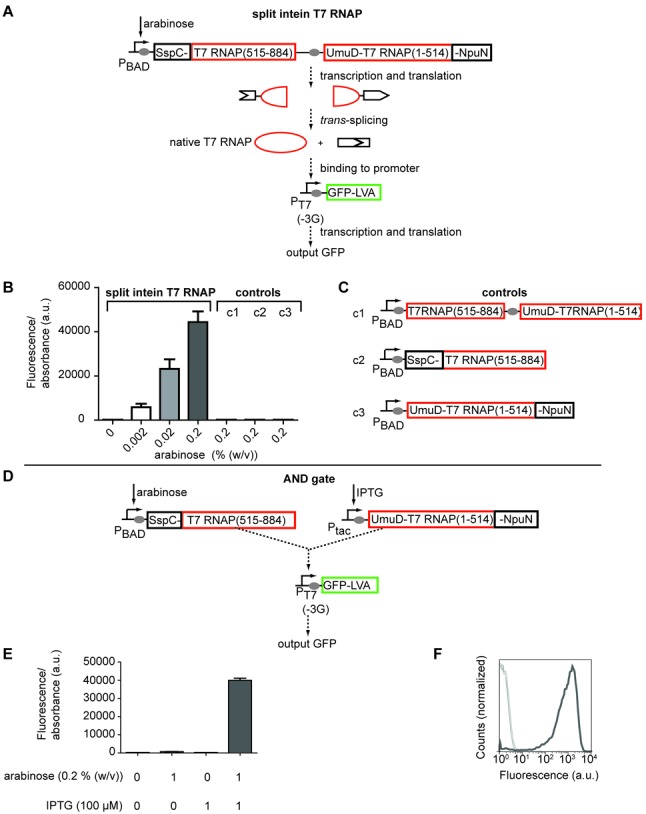
A split intein T7 RNAP for transcriptional AND-signal integration. (**A**) Schematic depiction of the designed construct and splicing. P_BAD_: arabinose inducible promoter; boxes: genes; grey ellipses: ribosomal binding sites; P_T7(−3G)_: T7 promoter with a G at position −3. (**B**) The construct depicted in (A) and control constructs (c1, c2, c3; (C)) were transformed into *E. coli*, together with the P_T7_-GFP reporter construct. The cells were grown for 8 h in the presence of the indicated amount of arabinose, and the fluorescence was measured. Mean and SD from three biological replicates. (**C**) Schematic depiction of control constructs. (**D**) Schematic depiction of an AND logic gate using the split intein T7 RNAP. (**E**) The construct depicted in (D) was transformed into *E. coli*, the cells were grown for 8 h in the presence of the indicated amounts of arabinose and IPTG, and their fluorescence was measured. Mean and SD from three biological replicates. (**F**) Flow cytometry data for all input states. The dark line is for the [11] sample.

The network scaffold used was described previously (14). Briefly, it consists of three compatible plasmids (pCOLA, pCDF, pET) each containing a multiple cloning site (MCS) for subcloning of the individual components and a set of transcriptional terminators. The plasmids contain different origins of replication (ori: ColA, CDF and pBR322) and antibiotic resistances (kanamycin, spectinomycin and ampicillin). The pCOLA plasmid constitutively expresses AraC and contains the P_BAD_ promoter (15). Therefore, the expression of genes cloned into this plasmid is induced by arabinose. The pET plasmid contains superfolder green fluorescent protein (GFP) (16) (with a C-terminal LVA degradation tag (17)) for the fluorescent readout. If only two plasmids were required (Figure 1A–C), the third plasmid (containing no genes in the MCS) was still transformed, in order to use the same conditions and medium in all experiments. The plasmids used for each experiment are given in Table 1.

**Table 1.** Plasmids used in this study and their GenBank accession numbers

Figure	Plasmid	Accession number
Figure 1A, B	pCOLA-AraC-pBAD-SspC-T7RNAP(515-884)-UmuD-T7RNAP(1-514)-NpuN	KM261828
	pCDF-MCS	KM261829
	pET-T7p(-3G)-SpacerA-GFP-LVA	KM261830
Figure 1B, C c1	pCOLA-AraC-pBAD-T7RNAP(515–884)-UmuD-T7RNAP(1-514)	KM261831
	pCDF-MCS	KM261829
	pET-T7p(-3G)-SpacerA-GFP-LVA	KM261830
Figure 1B, C c2	pCOLA-AraC-pBAD-SspC-T7RNAP(515-884)	KM261832
	pCDF-MCS	KM261829
	pET-T7p(-3G)-SpacerA-GFP-LVA	KM261830
Figure 1B, C c3	pCOLA-AraC-pBAD-UmuD-T7RNAP(1-514)-NpuN	KM261833
	pCDF-MCS	KM261829
	pET-T7p(-3G)-SpacerA-GFP-LVA	KM261830
Figure 1D-F	pCOLA-AraC-pBAD-SspC-T7RNAP(515-884)	KM261832
	pCDF-pTac-UmuD-T7RNAP(1-514)-NpuN	KM261834
	pET-T7p(-3G)-SpacerA-GFP-LVA	KM261830
Figure 2	pCOLA-AraC-pBAD-SspC-T7RNAP(515-884)-TetR-LVA	KM261835
	pCDF-J23106-TetO-UmuD-T7RNAP(1-514)-NpuN	KM261836
	pET-T7p(-12T)-SpacerA-GFP-LVA	KM261837

### *E. coli* strain

The *E. coli* strain used was described previously (14) and is a descendant of strain BW27783. In BW27783, the native araE promoter is replaced by a constitutive promoter (18). This results in a homogeneous cell population expressing genes under the control of the P_BAD_ promoter, with a graded response to arabinose. In addition, in the strain used, lacI (ECK0342) was replaced by a chloramphenicol resistance gene (strain MK01) (19) and tdk (ECK1233) was removed as previously described (20).

The bacterial cells were made electrocompetent (21) and aliquots were stored at −80°C. The three plasmids (pCOLA, pCDF and pET) were transformed simultaneously using a Bio-Rad gene pulser Xcell electroporator. Transformed bacteria were plated out on agar plates.

### Cloning

Polymerase chain reactions (PCRs), restriction digests and ligations were performed with standard protocols (21). Chemically competent TOP10 cells (Invitrogen) were used for subcloning. Plasmids were purified using a QIAprep Spin Miniprep Kit (QIAGEN). Restriction enzymes and T4 DNA ligase were purchased from New England BioLabs. KOD Hot Start polymerase was ordered from Novagen and oligonucleotides and chemicals were ordered from Sigma-Aldrich.

The intein amino acid sequence (22,23) was reverse-translated into DNA using the Sequence Manipulation Suite (24) and ordered as oligonucleotides for PCR-based assembly.

Sequences of all primers and nucleotides are given in Supplementary Table S1.

### UmuD-T7 RNAP(1-514)-NpuN

UmuD-T7 RNAP(1-514) was PCR amplified from the codon-optimised UmuD-T7 RNAP in pUC57 (14) with the primers pUC-f and T7(514)-NpuN_1_as. The intein NpuN was assembled from the oligonucleotides NpuN_2_s, NpuN_3_as, NpuN_4_s and NpuN_5_as. They were designed so that they overlap at ∼25 nucleotides and have ∼80 nucleotides of 5′ overhangs. NpuN_2_s and NpuN_3_as were annealed to each other and the overhangs were then ‘filled-in’ using KOD polymerase. The same was done for NpuN_4_s and NpuN_5_as. The two double-stranded DNA pieces were then joined by overlap extension PCR using NpuN_2_s and NpuN_5_as as primers. Finally, the UmuD-T7 RNAP(1-514) and NpuN were joined by overlap extension PCR using the primers pUC-f and NpuN_6_as.

### SspC-T7 RNAP(515-884)

T7 RNAP(515-884) was PCR amplified from the codon-optimised UmuD-T7 RNAP in pUC57 (14) with the primers T7(515)_s and T7_BamHI_as. The intein SspC was assembled from the oligonucleotides SspC_1_s and SspC_2_as. These oligonucleotides were annealed to each other and the overhangs were then ‘filled-in’ using KOD polymerase. T7 RNAP(515-884) and SspC were joined by overlap extension PCR using the primers SspC_3_s and T7_BamHI_as.

The genes were then cloned into the network scaffold as described previously (14).

### pCOLA-AraC-T7RNAP(515-884)-UmuD-T7RNAP(1-514)

For the control construct pCOLA-AraC-T7RNAP(515-884)-UmuD-T7RNAP(1-514), the inteins were removed by PCR amplifying pCOLA-AraC-SspC-T7RNAP(515-884)-UmuD-T7RNAP(1-514)-NpuN with the primers NoInt_insert_s and NoInt_insert_as and NoInt_back_s and NoInt_back_as. The two parts were joined by Gibson cloning (25).

### pCDF-lacI-Ptac-UmuD-T7RNAP(1-514)-NpuN

**Figure 2.**
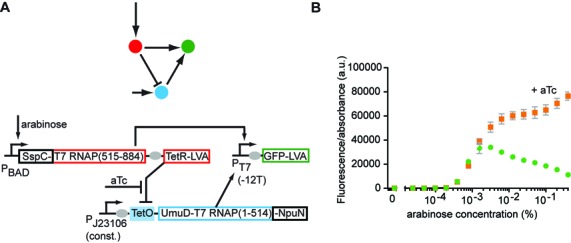
Application of the split intein T7 RNAP in a band-pass filter circuit. (**A**) Schematic view of the network and its implementation. P_J23106_: constitutive promoter; TetO: tet operator; aTc: anhydrotetracycline. (**B**) Bacteria carrying the network show a band-pass behaviour in an arabinose gradient (green). In the presence of aTc (orange), to prevent repression by TetR, the same circuit shows increasing fluorescence with increasing arabinose. Mean and SD from three biological replicates.

pCDF-J23106-TetO-UmuD-T7RNAP(1-514)-NpuN (for Figure 2) was cloned in the network scaffold (where lacI has previously been removed from the pCDF plasmid) (14). The MCS containing UmuD-T7 RNAP(1-514)-NpuN was PCR amplified using the primers AND_insert_s and AND_insert as. The original pCDF backbone including lacI and its constitutive promoter was PCR amplified from pCDF-1b (Novagen) using the primers AND_back_s and AND_back_as. The two parts were joined by Gibson cloning (25). Ptac was cloned between EcoRI and SacI using the oligonucleotides EcoRI_pTac_SacI_s and EcoRI_pTac_SacI_as.

### Media

Cloning steps used 1x Luria-Bertani medium (LB:10-g Bacto-tryptone, 5-g yeast extract, 10-g NaCl per 1 l), with appropriate antibiotics. For Figure 1 M9 medium (1x M9 minimal salts (Sigma-Aldrich), 1-mM MgSO_4_, 0.1-mM CaCl_2_, 0.4% (w/v) glucose, 0.1% (w/v) tryptone, 10-μg/ml thymidine, 50-μg/ml ampicillin, 15-μg/ml kanamycin and 25-μg/ml spectinomycin) was used. For Figure 2, ‘Stripe Medium’ (14) was used (SM: 1x LB plus 0.4% (w/v) glucose, 50-μg/ml ampicillin, 15-μg/ml kanamycin and 25-μg/ml spectinomycin).

### Fluorescence measurements

Fluorescence measurements were performed as described previously (14). Briefly, a single colony was picked for each biological replicate and grown overnight in 5-ml SM. The optical densities (OD) at 600 nm were measured and the cultures were diluted to OD 0.0015 in M9 medium (Figure 1) or SM (Figure 2) containing Isopropyl β-D-1-thiogalactopyranoside (IPTG) and arabinose when indicated. Subsequently, 120-μl of the diluted cultures were added to the wells of a 96-well plate and the absorbance at 600 nm and green fluorescence (excitation: 485 nm, emission: 520 nm) were measured every 6 min in a Tecan Infinite M200 plate reader for 8 h (Figure 1) or 6 h (Figure 2) at 37°C. Between readings, the plate was shaken for 220 s (orbital, 2 mm). The background signal of the medium was subtracted and the fluorescence was normalized for the number of cells by dividing by the absorbance. The background-corrected normalized fluorescence is shown at 8 h of growth (Figure 1) or 6 h (Figure 2). After 8 h of growth, 5 μl of the cultures were diluted into 1-ml phosphate buffered saline for flow cytometry analysis. A Becton–Dickinson FACScan flow cytometer with a 488 nm excitation laser, a 505 LP filter and a 530/28-nm BP filter was used. Cells were assayed in the low flow rate mode. For each sample, at least 50 000 events were collected. The data were analysed with the software FlowJo. Bacteria were gated in the forward scatter versus sideward scatter plot and the histograms of the green fluorescence are displayed in Figure 1F. Flow cytometry analysis was done in the CRG/UPF FACS Unit.

## RESULTS

### Design of split intein T7 RNAP

We chose a split intein based on the N-terminal Npu intein from *dnaE* in *Nostoc punctiforme* (NpuN, 102 amino acids) and the C-terminal Ssp intein from *dnaE* in *Synechocystic sp.* strain PCC6803 (SspC, 36 amino acids). This combination was chosen because of its high trans-splicing activity in *E. coli*, and its tolerance to amino acid substitutions in the splicing junction sequence (22,23). Two mutations were included (L25S in NpuN and P21R in SspC) as they have been shown to increase the splicing activity at 37°C in *E. coli* (23). The natural sequences at the splicing junctions of this split intein are ‘EY’, for the N-terminal extein and the canonical ‘CFN’ for the C-terminal extein. While the N-terminal splice junction is tolerant of noncanonical sequences (23), the +1 Cys residue in the C-extein serves as a nucleophile during the splicing process and is therefore essential (26). The +2 and +3 positions also contribute to the splicing efficiency, but indeed many residues support high splicing efficiencies (22,27). Because we did not want to introduce any mutation into the T7 RNAP, we looked for any natural occurrence of ‘CFN’ in the T7 RNAP sequence. As the exact sequence does not exist, we decided to split the T7 RNAP between amino acids 514 and 515, yielding ‘AF’ as the N-terminal extein junction sequence and ‘CFE’ as the C-terminal extein junction sequence; these residues form part of an α-helix.

After choosing the intein split site, we cloned the designed split intein T7 RNAP: SspC-T7 RNAP(515-884) (C-terminal part) and UmuD-T7 RNAP(1-514)-NpuN (N-terminal part). UmuD is a degradation tag (28,29) that we added to reduce the metabolic load imposed on the cells (14). It should be noted that the amino acid numbering above does not include the degradation tag.

### Functionality of split intein T7 RNAP

To test the functionality of split intein T7 RNAP (Figure 1) we put both parts as a single operon under the control of an arabinose inducible promoter (pBAD). When we induced the expression of the split intein T7 RNAP we indeed detected the expression of a T7 promoter-green fluorescent protein reporter (superfolder GFP (16) with a LVA degradation tag (17)) (Figure 1 A and B). This suggested that the *trans*-splicing correctly produced a functional full-length T7 RNAP. Moreover, none of the controls (Figure 1 B, C) gave any significant fluorescence (i.e. split T7 RNAP without intein, or either the C-terminal or N-terminal part of the split intein T7 RNAP alone).

Next, we cloned the C-terminal part of the split intein T7 RNAP (SspC-T7 RNAP(515-884)) under the control of an arabinose-inducible promoter and the N-terminal part (UmuD-T7 RNAP(1-514)-NpuN) under the control of an IPTG-inducible promoter (P_tac_). We confirmed that we built a functional AND gate since we only observed fluorescence upon adding both arabinose and IPTG (Figure 1 D–F) with a 57-fold induction compared to the highest control condition [1 0]. It should be noted that the output could be increased further, by using a stronger T7 promoter. We employed a mutant promoter P_T7(−3G)_ with a G at position −3 (instead of A) and ∼20% activity of the consensus T7 promoter (30), in order to keep the metabolic load to the cell minimal (14).

### Application in band-pass filter

To show the utility of this new part, we used it to build a band-pass filter (14,31–37), which expresses an output gene (GFP) only at intermediate input concentrations of chemical inducer and not at high or low concentrations. This function is a widely-used engineering target for synthetic biology (14,31–37) and the different mechanisms that can achieve it have recently been the subject of an exhaustive exploration (14). We previously showed that this function can be carried out by an incoherent feedforward loop type 4 (I4) (14) (Figure 2A). However, this network requires an AND-gate functionality and we reasoned that this might be achieved with a split intein system.

Figure 2A shows the implementation of the I4 bandpass filter topology using a split intein T7 RNAP as activator and TetR as repressor. At low inducer (arabinose) concentrations, we observe no fluorescence because SspC-T7 RNAP(515-884) is not expressed. At medium arabinose concentrations, we measure high fluorescence because SspC-T7 RNAP(515-884) and UmuD-T7 RNAP(1-514)-NpuN are expressed and are spliced to form an active T7 RNAP, that transcribes GFP-LVA. At high arabinose concentrations, the fluorescence decreases as the transcription of UmuD-T7 RNAP(1-514)-NpuN is repressed by TetR (Figure 2B). This repression can be prevented by adding anhydrotetracycline (aTc) to the medium, which causes derepression by binding to TetR. TetR can then no longer bind to its operator to inhibit transcription. This results in a continuous expression of UmuD-T7 RNAP(1-514)-NpuN and therefore in an increasing output GFP expression with increasing arabinose concentration in the presence of aTc (Figure 2B).

## DISCUSSION

Engineering Boolean logic gates is an important research area in synthetic biology (3–8,38–39). Reliably working orthogonal gates are essential if they are to be scaled and integrated to build larger and more complicated devices. However, only a limited number of transcriptional regulators and promoters have been described so far for utilization in AND gates. There is therefore a pressing need to expand the number of parts capable for transcriptional AND-logic. Here, we have demonstrated that split intein T7 RNAP can be used for transcriptional AND-signal integration. We divided T7 RNA polymerase into two expression domains and fused each to a split intein. Only when both domains are expressed does the split intein mediate protein trans-splicing, yielding a full-length T7 RNA polymerase that transcribes genes via a T7 promoter. We show the functionality of this part in an AND gate (Figure 1) and also employ it in a band-pass filter circuit (Figure 2). Importantly, the part has a low background and near digital behaviour, facilitating circuit engineering.

In contrast to the previously reported split T7 RNAP (8), where the resulting non-covalently assembled polymerase has reduced activity and decreased processivity (9), in the split intein T7 RNAP the active form is the native T7 RNAP. Therefore, any parameters determined for the wild-type T7 RNAP can be applied for the split intein T7 RNAP, which is very useful for network modelling and circuit design. Also, any functional modifications published for the wild-type T7 RNAP, and its promoter, can be directly transferred to the split intein T7 RNAP. These include mutations of the promoter to decrease expression (14) or mutations for orthogonal RNAP–promoter pairs (29).

Designing the split intein T7 RNAP was relatively straightforward; the first version we designed and tested is functional. We expect that the approach may also be successful when choosing different split sites in T7 RNAP. The main requirement on the split site is the presence of a nucleophile in the +1 position in the C-extein. In the split intein employed, this requires Cys residues (12 residues in T7 RNAP). However, in other split inteins this function can also be carried out by Ser (41 residues in T7 RNAP) or Thr (44 residues in T7 RNAP) (40). Although the other extein residues close to the splice junction also contribute to the splicing efficiency (27), a wide range of residues are accepted (22,27). As the spliced protein is the native form and not a non-covalently assembled protein, the position of the splicing site does not influence the activity of the spliced part. Controls are however necessary, to insure that the split parts are not active on their own (Figure 1 B and C).

Considering all the available options to design a split intein protein, this approach offers a lot of flexibility and is more efficient than having to rely on natural known split sites (8) or having to screen libraries for possible split sites (41). Since phage RNA polymerases share homology (e.g. T7, SP6 and T3 RNAPs), the split intein approach might be simply extended to these proteins (Supplementary Figure S1). Indeed, T3 RNAP contains ‘AF’ and ‘CFE’ split sites that are identical to T7 RNAP, while SP6 RNAP contains ‘AW’ and ‘CFE’. Although these split intein designs still need to be tested, the use of multiple RNAPs in parallel is routine in more complex network engineering (14).

We have demonstrated the utility of split intein T7 RNAP using the Npu and Ssp inteins. A wide range of other natural—as well as engineered—split inteins have been characterized (42), some of them as short as six amino acids (mini-inteins) (43) or consisting of three-pieces (44). Several of these split inteins have been shown to be orthogonal to each other, i.e. they do not show cross-reactivity (45). This opens up the possibility of employing several split intein systems in the same circuit. For example, two versions of split intein T7 RNAP each using a different split intein could be used to operate two independent AND gates on the same T7 promoter.

Synthetic biology is in constant need for robust parts to implement ever more sophisticated circuits. The split intein approach has so far been applied to zinc finger and Transcriptional activator-like effector (TALE) transcription factors for logic computations in mammalian cells (12,13). We showed here that the approach is also working for split intein T7 RNAP in *E. coli* and can be applied to proteins that are not as modular as the above mentioned transcription factors. We therefore believe that this new part will prove useful and widely applicable in different synthetic circuits.

## SUPPLEMENTARY DATA

Supplementary Data are available at NAR Online.

SUPPLEMENTARY DATA

## ACCESSION NUMBERS

GenBank accession codes: KM261828, KM261829, KM261830, KM261831, KM261832, KM261833, KM261834, KM261835, KM261836 and KM261837.
