# Supplementary material for: A split intein T7 RNA polymerase for transcriptional AND-logic
Source: Nucleic Acids Res. 2014 Sep 27;42(19):12322–8. doi: 10.1093/nar/gku884 (PMC4231753; doi:10.1093/nar/gku884)
Supplement: SUPPLEMENTARY DATA [file supp_42_19_12322__index.html]

A split intein T7 RNA polymerase for transcriptional AND-logic — A split intein T7 RNA polymerase for transcriptional AND-logic — SUPPLEMENTARY DATA 

# A split intein T7 RNA polymerase for transcriptional AND-logic

## SUPPLEMENTARY DATA

**Files in this Data Supplement:**

- SUPPLEMENTARY DATA
