# Supplementary material for: A split intein T7 RNA polymerase for transcriptional AND-logic
Source: Nucleic Acids Res. 2014 Sep 27;42(19):12322–8. doi: 10.1093/nar/gku884 (PMC4231753; doi:10.1093/nar/gku884)
Supplement: SUPPLEMENTARY DATA [file supp_gku884_nar-02175-h-2014-File004.pdf]

Supplementary Data

Name	Sequence 5' -> 3'
pUC_f	GCATCAGAGCAGATTGTACTGAG
T7(514)-NpuN_1_as	CATATTCCACGGTCAGAATTTCGGTTTCATAGCTCAGGCAAAACGCCAGGAAGCAAAAC
NpuN_2_s	CGAAATTCTGACCGTGGAATATGGCAGCCTGCCGATTGGCAAAATTGTGGAAAAACGCATTGAATGCACCGTGTATAGCGTGGATAACAACGGCAACATTTATACCCAG
NpuN_3_as	CGAATCAGGCTGCCATCTTCCAGGCAATATTCAAACACTTCCTGTTCGCCGCGATCATGCCACTGCGCCACCGGCTGGGTATAAATGTTGCCGTTGTTATC
NpuN_4_s	CTGGAAGATGGCAGCCTGATTCGCGCGACCAAAGATCATAAATTTATGACCGTGGATGGCCAGATGCTGCCGATTGATG
NpuN_5_as	GCATGATCGGATCCTTAGTTCGGCAGGTTATCCACGCGCATCAGATCCAGTTCGCGTTCAAAAATTTCATCAATCGGCAGCATCTG
NpuN_6_as	GCATGATC GGATCC TTAGTTCG
SspC_1_s	GATCTAGCATATGGTGAAAGTGATTGGCCGCCGCAGCCTGGGCGTGCAGCGCATTTTTGATATTGGCCTGCGCCAGG
SspC_2_T7(514)_as	GAACGCCGGCATATTCGAAACAGTTCGCCGCAATCGCGCCGTTCGCCAGCAGAAAGTTATGATCCTGGCGCAGGCCAATATC
T7(515)_s	TGTTTCGAATATGCCGGCGT
T7_BamHI_as	CTGCAGGGGATCCTTATTACGCA
SspC_3_s	GATCTAGCATATGGTGAAAGTGATTGG
NoInt_insert_s	TAAGAAGGAGATATACATATGTGTTTCGAATATGCCGGCGTTC
NoInt_insert_as	GAGCGGCGCCGGATCCTTAAAACGCCAGGAAGCAAAAC
NoInt_back_s	GTTTTGCTTCCTGGCGTTTTAAGGATCCGGCGCCGCTC
NoInt_back_as	GCCGGCATATTCGAAACACATATGTATATCTCCTTCTTAAAGTTAAACAAAAT
AND_back_s	CCTTTTTGCGTTTCAGATCTACCGGTAAACCAGCAATAGACAT
AND_back_as	CCGTCGTTTTACAACTCGAGGTCGCATAAGGGAGAGCGTC
AND_insert_s	GACGCTCTCCCTTATGCGAC CTCGAGTTGTAAAACGACGGCCAG
AND_insert_as	CTATTGCTGGTTTACCGGT ACCGGTAGATCTGAAACGCAAAAAG
EcoRI-pTac-SacI_s	AATTCGAGCTGTTGACAATTAATCATCGGCTCGTATAATGTGTGGAATTGTGAGCGGATAACAATTTCACACGAGCT
EcoRI-pTac-SacI_as	CGTGTGAAATTGTTATCCGCTCACAATTCCACACATTATACGAGCCGATGATTAATTGTCAACAGCTCG

Supplementary Table 1. Sequences of primers and oligonucleotides used for cloning.

```
>T7_RNAP

MNTINIAKNDFSDIELAAIPFNTLADHYGERLAREQLALEHESYEMGEARFRKMFERQLKAGEVADNAAAKPLITTLLPKMIARI
NDWFEEVKAKRGKRPTAFQFLQEIKPEAVAYITIKTTLACLTSADNTTVQAVASAIGRAIEDEARFGRIRDLEAKHFKKNVEEQL
NKRVGHVYKKAFMQVVEADMLSKGLLGGEAWSSWHKEDSIHVGVRCIEMLIESTGMVSLHRQNAGVVGQDSETIELAPEYAEAIA
TRAGALAGISPMFQPCVVPPKPWTGITGGGYWANGRRPLALVRTHSKKALMRYEDVYMPEVYKAINIAQNTAWKINKKVLAVANV
ITKWKHCPVEDIPAIEREELPMKPEDIDMNPEALTAWKRAAAAVYRKDKARKSRRISLEFMLEQANKFANHKAIWFPYNMDWRGR
VYAVSMFNPQGNDMTKGLLTLAKGKPIGKEGYYWLKIHGANCAGVDKVPFPERIKFIEENHENIMACAKSPLENTWWAEQDSPFC
FLAFCFEYAGVQHHGLSYNCSLPLAFDGSCSGIQHFSAMLRDEVGGRAVNLLPSETVQDIYGIVAKKVNEILQADAINGTDNEVV
TVTDENTGEISEKVKLGTKALAGQWLAYGVTRSVTKRSVMTLAYGSKEFGFRQQVLEDTIQPAIDSGKGLMFTQPNQAAGYMAKL
IWESVSVTVVAAVEAMNWLKSAAKLLAAEVKDKKTGEILRKRCAVHWVTPDGFPVWQEYKKPIQTRLNLMFLGQFRLQPTINTNK
DSEIDAHKQESGIAPNFVHSQDGSHLRKTVVWAHEKYGIESFALIHDSSFGTIPADAANLFKAVRETMVDTYESCDVLADFYDQF
ADQLHESQLDKMPALPAKGNLNLRDILESDFAFA*

>T3_RNAP

MNIIENIEKNDFSEIELAAIPFNTLADHYGSALAKEQLALEHESYELGERRFLKMLERQAKAGEIADNAAAKPLLATLLPKLTTR
IVEWLEEYASKKGRKPSAYAPLQLLKPEASAFITLKVILASLTSTNMTTIQAAAGMLGKAIEDEARFGRIRDLEAKHFKKHVEEQ
LNKRHGQVYKKAFMQVVEADMIGRGLLGGEAWSSWDKETTMHVGIRLIEMLIESTGLVELQRHNAGNAGSDHEALQLAQEYVDVL
AKRAGALAGISPMFQPCVVPPKPWVAITGGGYWANGRRPLALVRTHSKKGLMRYEDVYMPEVYKAVNLAQNTAWKINKKVLAVVN
EIVNWKNCPVADIPSLERQELPPKPDDIDTNEAALKEWKKAAAGIYRLDKARVSRRISLEFMLEQANKFASKKAIWFPYNMDWRG
RVYAVPMFNPQGNDMTKGLLTLAKGKPIGEEGFYWLKIHGANCAGVDKVPFPERIAFIEKHVDDILACAKDPINNTWWAEQDSPF
CFLAFCFEYAGVTHHGLSYNCSLPLAFDGSCSGIQHFSAMLRDEVGGRAVNLLPSETVQDIYGIVAQKVNEILKQDAINGTPNEM
ITVTDKDTGEISEKLKLGTSTLAQQWLAYGVTRSVTKRSVMTLAYGSKEFGFRQQVLDDTIQPAIDSGKGLMFTQPNQAAGYMAK
LIWDAVSVTVVAAVEAMNWLKSAAKLLAAEVKDKKTKEILRHRCAVHWTTPDGFPVWQEYRKPLQKRLDMIFLGQFRLQPTINTL
KDSGIDAHKQESGIAPNFVHSQDGSHLRMTVVYAHEKYGIESFALIHDSFGTIPADAGKLFKAVRETMVITYENNDVLADFYSQF
ADQLHETQLDKMPPLPKKGNLNLQDILKSDFAFA*

>SP6_RNAP

MQDLHAIQLQLEEEMFNGGIRRFEADQQRQIAAGSESDTAWNRRLLSELIAPMAEGIQAYKEEYEGKKGRAPRALAFLQCVENEV
AAYITMKVVMDMLNTDATLQAIAMSVAERIEDQVRFSKLEGHAAKYFEKVKKSLKASRTKSYRHAHNVAVVAEKSVAEKDADFDR
WEAWPKETQLQIGTTLLEILEGSVFYNGEPVFMRAMRTYGGKTIYYLQTSESVGQWISAFKEHVAQLSPAYAPCVIPPRPWRTPF
NGGFHTEKVASRIRLVKGNREHVRKLTQKQMPKVYKAINALQNTQWQINKDVLAVIEEVIRLDLGYGVPSFKPLIDKENKPANPV
PVEFQHLRGRELKEMLSPEQWQQFINWKGECARLYTAETKRGSKSAAVVRMVGQARKYSAFESIYFVYAMDSRSRVYVQSSTLSP
QSNDLGKALLRFTEGRPVNGVEALKWFCINGANLWGWDKKTFDVRVSNVLDEEFQDMCRDIAADPLTFTQWAKADAPYEFLAWCF
EYAQYLDLVDEGRADEFRTHLPVHQDGSCSGIQHYSAMLRDEVGAKAVNLKPSDAPQDIYGAVAQVVIKKNALYMDADDATTFTS
GSVTLSGTELRAMASAWDSIGITRSLTKKPVMTLPYGSTRLTCRESVIDYIVDLEEKEAQKAVAEGRTANKVHPFEDDRQDYLTP
GAAYNYMTALIWPSISEVVKAPIVAMKMIRQLARFAAKRNEGLMYTLPTGFILEQKIMATEMLRVRTCLMGDIKMSLQVETDIVD
EAAMMGAAAPNFVHGHDASHLILTVCELVDKGVTSIAVIHDSSFGTHADNTLTLRVALKGQMVAMYIDGNALQKLLEEHEERWMV
DTGIEVPEQGEFDLNEIMDSEYVFA*
```

Supplementary Fig. 1. The sequences of T7, SP6 and T3 RNA polymerases (RNAPs) are homologous, such that the split intein approach might be extended to the latter two. T3 RNAP contains identical "AF" and "CFE" splicing sites which were shown to work in this study, in the context of T7 RNAP (highlighted in red and blue, respectively). SP6 RNAP contains almost identical "AW" and "CFE" sites. Both the T3 and SP6 designs are theoretical and would need to be tested for splicing function in *E. coli*.
